# Transcatheter embolization of an endoleak through a vascular plug implanted in the left common iliac artery during aortouni-iliac endovascular aortic repair: A case report

**DOI:** 10.1016/j.radcr.2026.06.034

**Published:** 2026-06-29

**Authors:** Ryuta Okuhira, Akira Ikoma, Ryosuke Mimura, Atsufumi Kamisako, Tetsuo Sonomura, Hiroshi Doi

**Affiliations:** Department of Radiology, Wakayama Medical University, 811-1 Kimiidera, Wakayama City, Wakayama 641-8510, Japan

**Keywords:** Embolization, Endoleak, Vascular plug, Endovascular aortic repair

## Abstract

A 90-year-old woman underwent aortouni-iliac endovascular aortic repair and simultaneous embolization of the left common iliac artery using a vascular plug for an impending rupture of an abdominal aortic aneurysm. The aortic aneurysm diameter gradually increased after the procedure, and an endoleak through the vascular plug in the left common iliac artery was observed by contrast-enhanced computed tomography. Using a highly penetrating microsystem to penetrate the mesh of the vascular plug, metallic coils were placed inside and around the vascular plug. Using a triple coaxial system, the proximal portion of the left common iliac artery and the endoleak cavity of the aneurysm were embolized with 20% n-butyl-2-cyanoacrylate‒Lipiodol mixture via collateral arteries. The endoleak disappeared after transcatheter embolization. To our knowledge, this is the first reported case in which transcatheter embolization with metallic coils using a highly penetrating microsystem was performed to treat an endoleak through a vascular plug following endovascular aortic repair with an aortouni-iliac device and simultaneous embolization of the contralateral common iliac artery.

## Introduction

Endovascular aortic repair (EVAR) using an aortouni-iliac (AUI) device may be used to treat abdominal aortic aneurysms if there are anatomic constraints and limited availability of grafts in emergency situations [1,2]. AUI-EVAR does not require cannulation into the contralateral gate and can isolate aneurysms more quickly than EVAR using bifurcated device, which may be advantageous in emergency situations [3]. The contralateral common iliac artery (CIA) is usually embolized with metallic coils or a vascular plug, and femoro-femoral crossover bypass is performed to ensure peripheral blood flow on the contralateral side [[4], [5], [6]]. However, AUI-EVAR is associated with a higher incidence of postoperative endoleaks/complications and higher mortality compared with EVAR using bifurcated device [7,8].

Here, we report the first successful case of transcatheter embolization of an endoleak through the left CIA, which had been embolized by a vascular plug during AUI-EVAR for an impending rupture of an abdominal aortic aneurysm.

## Case report

A 90-year-old woman presented to our hospital with acute abdominal pain. She underwent emergency EVAR for an impending rupture of an abdominal aortic aneurysm measuring 63 mm in maximum diameter. Because the left CIA was severely calcified, she underwent AUI-EVAR with deployment of an Endurant II AUI device (32 × 102 mm, Medtronic, Santa Rosa, CA) from the infrarenal aorta to the right CIA, along with placement of a GORE Excluder contralateral leg component (16 mm × 9.5 cm, W. L. Gore & Associates, Newark, DE) as a right iliac extension. Femoro-femoral crossover bypass was performed, and the left CIA was embolized using a 14-mm Amplatzer Vascular Plug II (AVP II; Abbott Medical, Santa Clara, CA). Thereafter, the aortic aneurysm diameter gradually increased. Three years and 4 months after the surgery, an aortic cuff (28.5 mm × 3.3 cm, GORE Excluder Aortic Extender, W. L. Gore & Associates) was placed on the proximal side of the stent graft owing to suspicion of a type Ia endoleak. Subsequently, contrast-enhanced computed tomography revealed an endoleak through the vascular plug in the left CIA (Fig. 1). The patient was referred to our department for transcatheter embolization of the endoleak. A 4 Fr sheath (Terumo, Tokyo, Japan) was inserted via the left superficial femoral artery under ultrasound guidance. Retrograde left iliac arteriography revealed blood flow into the aneurysm through the vascular plug (Fig. 2A). Using a microguidewire with a heavy tip load (Astato xs 9-12, Asahi Intecc, Aichi, Japan), a highly penetrating microcatheter (Corsair Armet, Asahi Intecc) was inserted into the plug through the mesh of the plug (Fig. 2B). Because it was impossible to access the proximal portion of the left CIA, the parts inside and around the vascular plug were embolized with metallic coils. However, the endoleak proximal to the vascular plug remained after coil embolization, and a type II endoleak via the left iliolumbar artery branching from the left internal iliac artery and the left fourth lumbar artery was found (Fig. 3). Using a triple coaxial system, a microcatheter was inserted into the endoleak cavity and then into the proximal portion of the left CIA via the collateral pathway (Fig. 4A). The proximal portion of the left CIA, the endoleak cavity, and the left lumbar artery were embolized with 20% n-butyl-2-cyanoacrylate (NBCA; Histoacryl, B. Braun, Melsungen, Germany)—Lipiodol (Guerbet, Villepinte, France) mixture (Fig. 4B). The endoleak disappeared after embolization (Fig. 5).

**Fig. 1:**
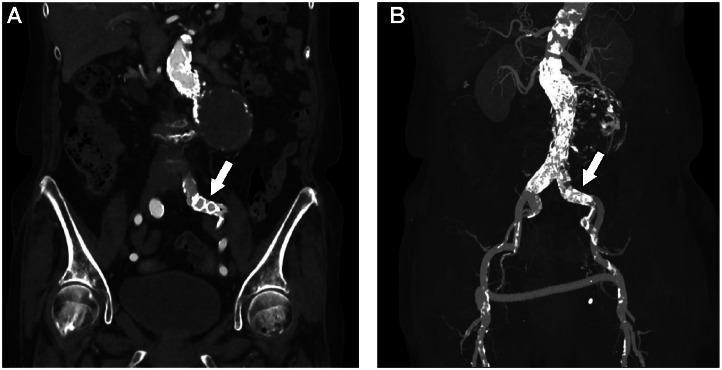
(A) Pre-embolization coronal contrast-enhanced computed tomography showing the contrast material within the vascular plug (arrow) placed in the left common iliac artery and the endoleak flowing proximally beyond the vascular plug and (B) Maximum intensity projection image produced from the pre-embolization contrast-enhanced computed tomography showing major calcification of the left common iliac artery (arrow).

**Fig. 2:**
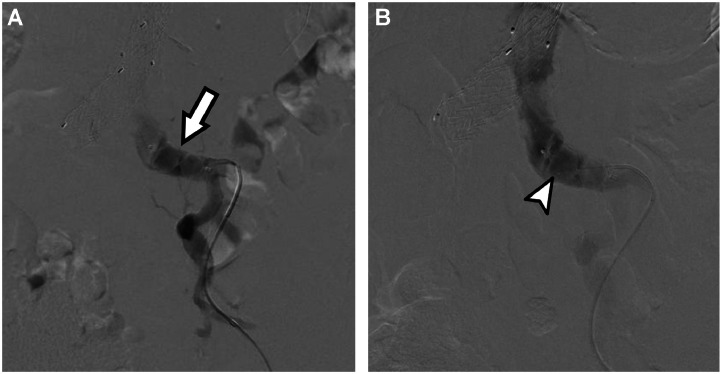
(A) Left iliac arteriography during treatment showing the endoleak through the vascular plug (arrow) placed in the left common iliac artery and (B) Image showing the highly penetrating microcatheter inserted into the vascular plug using a microguidewire with a heavy tip load (arrowhead indicates the microcatheter tip).

**Fig. 3:**
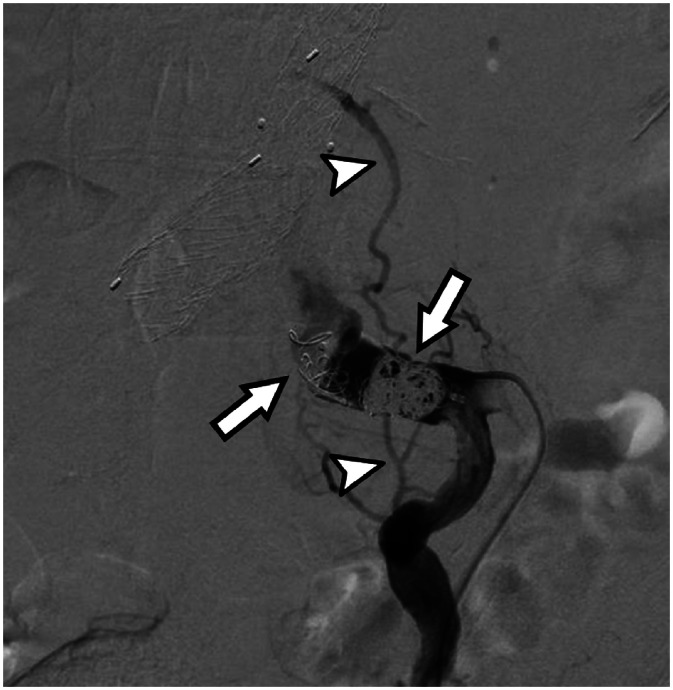
Angiography after placing the metallic coils (arrows) inside and around the vascular plug showing the residual endoleak and an apparent type II endoleak through the left iliolumbar artery and lumbar artery (arrowheads).

**Fig. 4:**
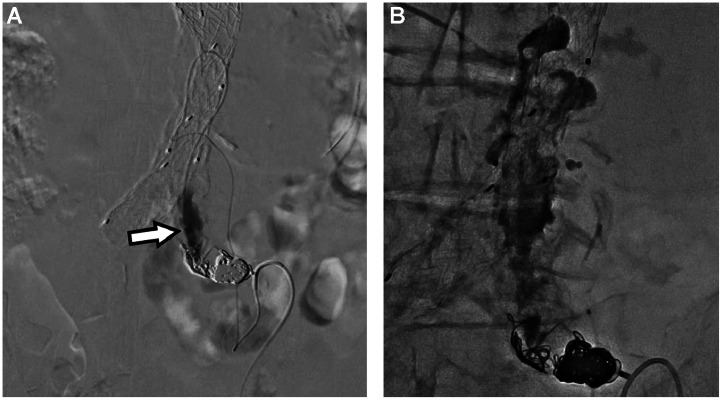
(A) Imaging showing the tip of the microcatheter (arrow) inserted into the proximal portion of the left common iliac artery through the left iliolumbar artery, lumbar artery, and aneurysm using a triple coaxial system and (B) Intraoperative fluoroscopic image showing n-butyl-2-cyanoacrylate‒Lipiodol mixture injected into the proximal portion of the left common iliac artery, endoleak cavity, and left lumbar artery.

**Fig. 5:**
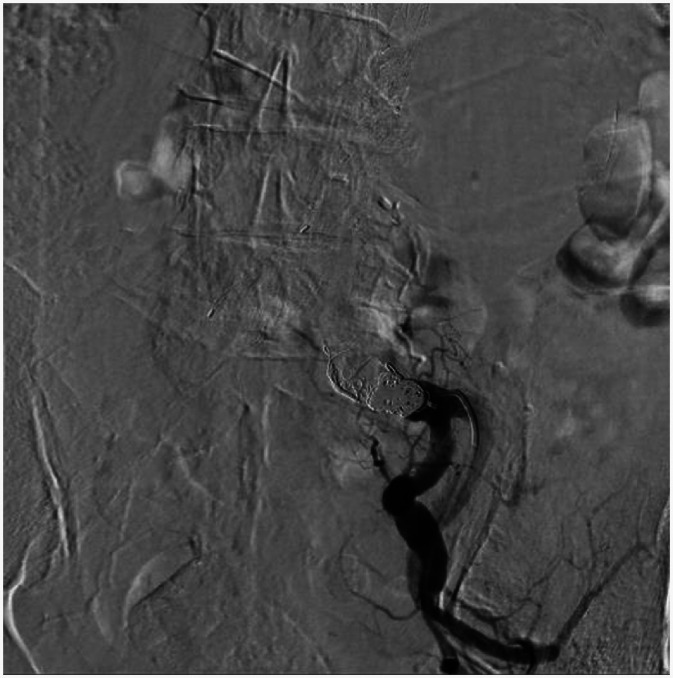
Left iliac arteriography immediately after embolization showing disappearance of the endoleak.

## Discussion

AUI-EVAR is associated with complications common to bifurcated EVAR, such as endoleaks, graft migration, and limb occlusion. Additionally, complications specific to the concomitant femoro-femoral crossover bypass must be considered, including groin access-site complications and bypass graft occlusion or infection. Among these complications, endoleak is one of the most common causes of reintervention after EVAR; the reported 1-year endoleak rate after AUI-EVAR is approximately 7% [2]. However, the specific incidence of type Ic endoleak through a contralateral CIA embolized with coils or a vascular plug remains unclear. To our knowledge, such type Ic endoleaks have been rarely reported in the literature, highlighting the clinical importance of our findings.

Vascular plugs are devices used for short-segment embolization, including embolization of branch arteries, during branched stent grafting and embolization of the left subclavian artery during thoracic EVAR [[9], [10], [11], [12], [13], [14]]. Compared with metallic coils, which are commonly used for branch artery embolization, vascular plugs reduce the procedure time and increase the cost-effectiveness [[12], [13], [14]]. Additionally, vascular plugs are generally accepted as an embolic material for embolization of the contralateral CIA during AUI-EVAR. However, the occluded segment may recanalize after embolization with a vascular plug alone [[15], [16], [17]]. In this case, we performed successful transcatheter embolization to treat the endoleak via the CIA, which had been embolized with a vascular plug.

We speculated that the endoleak through the CIA, which had been embolized with a vascular plug, was caused by inadequate contact between the plug and the severely calcified arterial wall. Therefore, the blood flow was not sufficiently blocked and the embolized site was not fully thrombosed. In cases with severe calcification of the arterial wall and/or high tortuosity of the artery, embolization with a vascular plug alone is not appropriate, and embolization with metallic coils or a combination of a vascular plug and metallic coils should be considered [18]. Embolization of the contralateral iliac artery using a vascular plug may be a risk factor for endoleak in cases where an AUI device is used due to the poor condition of the iliac artery.

In this case, by using a highly penetrating microsystem, we could embolize the parts inside and around the vascular plug with metallic coils [19]. In addition, by using a triple coaxial system, the proximal portion of the vascular plug could be embolized with NBCA‒Lipidiol.

## Conclusion

We observed a rare endoleak through the left CIA, which had been embolized with a vascular plug during AUI-EVAR. A highly penetrating microsystem was used for embolization with metallic coils through the vascular plug mesh, and a triple coaxial system was inserted via the collateral arteries for embolization using NBCA‒Lipiodol.

## Patient consent

A written informed consent was obtained from the patient for the publication of this case report.
